# Endotracheal cuff pressure: role of tracheal size and cuff volume

**DOI:** 10.1186/cc9567

**Published:** 2011-03-11

**Authors:** PL Lichtenthal, UB Borg

**Affiliations:** 1 University of Arizona, Tucson, AZ, USA; 2 Covidien, Boulder, CO, USA

## Introduction

To resolve conflicting issues of volume/pressure relationships in endotracheal (ETT) cuffs, we examined this using an animal model. Sengupta and colleagues concluded that cuff volumes were fairly consistent despite varying tracheal and ETT sizes [1]. Hoffman and colleagues concluded that the volume/pressure relationships in ETT cuffs are linear and that additional air volume above that necessary to reach safe sealing pressure would not result in a precipitous increase in pressure [2].

## Methods

In a study approved by the Animal Care and Use Committee, excised canine tracheas with four diameters (18, 20, 23 and 26 mm) were intubated with six different 7.5 mm ETTs from different manufacturers (Hi-Lo, TaperGuard and Hi-Lo Intermediate, Tyco Healthcare, Pleasanton, CA, USA; Blue Line SACETT Portex, Smith Medical, Keene, NH, USA; Teleflex ISIS HVT, Research Triangle Park, NC, USA; MicroCuff, Kimberly Clark, Roswell, GA, USA). Cuff pressure was determined with a pressure transducer located at the same level as the cuff and connected via the air-filled inflation line. The cuffs were inflated stepwise adding 1 ml of air per step.

## Results

The volume/pressure relationship for all cuffs is initially dependent on the resting volume of the cuff. Once the cuff pressure is equal to the force of the tracheal durometer, the cuff pressure increases linearly, reflecting the compliance of the trachea. This occurs at a cuff pressure of 30 cmH_2_O. In high-volume low-pressure cuffs (Hi-Lo, SACETT, ISIS) the inflation volume was greater compared with low-volume low-pressure cuffs (TaperGuard, Hi-Lo Intermediate). The polyurethane cuff (PU, MicroCuff) exhibited a unique volume/pressure relationship.

## Conclusions

The tracheal diameter influences the volume necessary to reach a certain cuff pressure with the same-size cuff, contrary to the findings of Sengupta and colleagues [1]. The type of cuff, high-volume low-pressure versus low-volume low-pressure, greatly influences the behavior of the cuff pressure. The high-volume low-pressure cuffs required the largest inflation volume. The type of material changes the behavior of the volume/pressure relationship. A PU cuff has a more nonlinear volume/pressure relationship compared with polyvinylchloride cuffs since PU is less distensible. It should be noted that the commonly recommended inflation pressure (25 to 30 cmH_2_O) [3] was the point at which the steep linear rise in pressure was seen with small increments of added inflation volume. In conclusion, we have demonstrated that ETT cuff pressure is multifactorial including cuff volume, material and tracheal diameter.
